# Radiomics-based machine learning for automated detection of Pneumothorax in CT scans

**DOI:** 10.1371/journal.pone.0314988

**Published:** 2024-12-09

**Authors:** Hanieh Alimiri Dehbaghi, Karim Khoshgard, Hamid Sharini, Samira Jafari Khairabadi, Farhad Naleini

**Affiliations:** 1 Department of Medical Physics, University of Medical Sciences, Kermanshah, Iran; 2 Department of Biomedical Engineering, University of Medical Sciences, Kermanshah, Iran; 3 Student Research Committee, University of Medical Sciences, Kermanshah, Iran; 4 Clinical Research Development Center, Imam Reza Hospital, University of Medical Sciences, Kermanshah, Iran; Stanford University School of Medicine, UNITED STATES OF AMERICA

## Abstract

The increasing complexity of diagnostic imaging often leads to misinterpretations and diagnostic errors, particularly in critical conditions such as pneumothorax. This study addresses the pressing need for improved diagnostic accuracy in CT scans by developing an intelligent model that leverages radiomics features and machine learning techniques. By enhancing the detection of pneumothorax, this research aims to mitigate diagnostic errors and accelerate the process of image interpretation, ultimately improving patient outcomes. Data used in this study was extracted from the medical records of 175 patients with suspected pneumothorax. The collected images were preprocessed in Matlab software. Radiomics features were extracted from each image and finally, the machine learning models were implemented on these features. The used machine learning algorithms are Gradient Tree Boosting (GBM), eXtreme Gradient Boosting (XGBoost), and Light GBM. To evaluate the performance of models, various evaluation criteria such as precision, accuracy, specificity, sensitivity, F1 score, Area Under the Receiver Operating Characteristic (ROC) Curve (AUC), and misclassification were calculated. According to the calculated evaluation criteria, in terms of accuracy, the Gradient Boosting Machine (GBM) model achieved the highest performance with an accuracy of 98.97%, followed closely by the XGBoost model at 98.29%. For precision, the GBM model outperformed the other models, recording a precision value of 99.55%. Regarding sensitivity, all three models—GBM, XGBoost, and LightGBM (LGBM)—demonstrated strong performance, with sensitivity values of 99%, 99%, and 100%, respectively, indicating minimal variation among them. The artificial intelligence models used in this study have significant potential to enhance patient care by supporting radiologists and other clinicians in the diagnosis of pneumothorax. These models can facilitate the prioritization of positive cases, expedite evaluations, and ultimately improve patient outcomes.

## 1 Introduction

Pneumothorax is the abnormal accumulation of air in the pleural cavity, between the parietal pleura and visceral pleura [1]. Pneumothorax may occur following trauma and non-traumatic causes such as injury to the chest, medical interventions, or due to an underlying lung disease such as Chronic Obstructive Pulmonary Disease (COPD) [2]. This complication can gradually progress to tension pneumothorax and become an emergency, so its timely diagnosis is essential [3,4]. Pneumothorax is also known as one of the important complications of Corona Virus Disease 2019 (COVID-19) and has increased the hospitalization rate [5,6]. This complication can lead to respiratory failure, cardiac arrest, and even death in severe cases [5], so it is a potentially life-threatening condition that requires prompt diagnosis and immediate treatment [1]. Standing chest radiography is usually the first imaging modality that is requested to diagnose pneumothorax, but it is difficult to detect small pneumothoraxes in radiographic images. In more than 30% of pneumothorax cases, there is a possibility of missing the diagnosis [7]. In general, it is difficult to interpret chest X-ray images to diagnose pneumothorax. The images may have multiple superimposed structures, the patterns of different chest diseases have different appearances, sizes, and positions on the radiographic images, and the changing positions of the patients taking X-ray images may cause distortions in the image [5]. In comparison with radiography, computed tomography (CT) provides several advantages. Occult pneumothorax occurs in up to 50% of traumatic pneumothoraxes and is not detected on chest X-ray. For this reason, chest CT has long been recommended when pneumothorax is suspected [8–11]. A CT scan can also help doctors determine the size of the pneumothorax more accurately and make treatment decisions [12].

For over three decades, chest CT scans have stood as the established "gold standard" for pneumothorax diagnosis [13–15], However, the prompt interpretation of these scans remains a challenging aspect [1]. The urgency of imaging procedures in the emergency department revolves around identifying patients necessitating swift diagnosis and immediate intervention [16], with pneumothorax being a prime example. Radiology departments grapple with the daily interpretation of a substantial volume of medical images from diverse modalities, escalating the workload for radiologists and posing a risk to accurate diagnoses. In the routine practice of radiology, radiologists are tasked with reading and interpreting medical images originating from various modalities. Typically, these professionals are required to conduct comprehensive analyses and evaluations of these images within tight timeframes. Nevertheless, as a result of advancements in modern medical technologies, the volume of imaging data is rapidly escalating. For instance, CT examinations now involve thinner slices compared to historical practices [17]. Moreover, not all imaging departments have on-site coverage by radiologists 24 hours a day. The confluence of advancements in imaging and computer science has given rise to the burgeoning potential of artificial intelligence (AI) applications, particularly in various imaging tasks [18]. Notably, the continuous evolution of AI algorithms, such as machine learning, has ushered in possibilities for expedited diagnosis and treatment, particularly in high-stakes settings like emergency rooms. The availability of robust Python programming language libraries has significantly facilitated the integration and utilization of machine learning techniques in radiology departments [19].

Artificial intelligence, particularly machine learning, has garnered significant attention across various domains within health and medicine. Notably, its application in the Emergency Department (ED) and triage has become crucial for swift and efficient diagnosis and treatment. The escalating number of ED consultations poses challenges to existing patient management methods, prompting the exploration of innovative solutions. Integrating artificial intelligence techniques, including machine learning and deep learning algorithms, stands out as a promising approach to enhance workflow and elevate patient care standards in EDs. The adoption of intelligent techniques holds the potential to mitigate human error, streamline processes, optimize resource allocation, and expedite schedules. Furthermore, machine learning systems frequently exhibit comparable or superior accuracy compared to clinical staff, underscoring their utility in healthcare settings [20].

Hence, the creation of an artificial intelligence model for pneumothorax diagnosis serves to assist radiologists in identifying, quantifying, and analyzing changes in lesion size over time. Such models prove particularly valuable in rural areas where access to doctors is limited [21].

Machine learning systems exhibit the capability for predicting and promptly diagnosing various pathologies within the emergency department, enhancing the effectiveness of treatment strategies. This potential aids in preventing the progression of diseases and mitigating the occurrence of untoward complications.

Machine learning stands as a captivating domain within computer science and engineering, acknowledged as a subset of artificial intelligence. Its capacity to discern intricate relationships or patterns from input data enables accurate decision-making—a facet reminiscent of human intelligence [22]. In the realm of medical imaging, machine learning emerges as a potent tool for automating analysis and diagnosis. Its application holds the promise of alleviating the workload on radiologists in the practice of radiology [17].

In a 2020 study by Sebastian Röhrich et al., [23] a deep residual UNet was used for the automatic classification of pneumothorax at the volume level (labeling a volume whether pneumothorax is present or not). In this study, the AUC criterion for the automatic detection of pneumothorax at the volume level was calculated as 0.97 and the average accuracy as 0.95.

Since deep learning requires stronger hardware and software requirements, in this study we tried to achieve better results by implementing machine learning algorithms and using minimal resources.

Over the past few decades, substantial advancements in the field of medical image analysis have enabled the extraction of quantitative features that may not be visually apparent [24,25].This process, known as radiomics, involves capturing tissue and lesion characteristics, including heterogeneity and shape. These radiomics features play a crucial role in predicting present target variables, such as the presence or absence of a disease or tumor type, as well as future variables like treatment response or time to recurrence [25].

Radiomics serves as an automated approach for generating features, extracting numerous quantitative phenotypes (radiomics features) from radiological images [25,26]. Machine Learning (ML) algorithms can then undergo training to identify associations between these radiomics features and patient diagnoses [27].

To the best of our knowledge, there has been no published study that presents a comprehensive framework integrating machine learning algorithms with radiomics features specifically for the diagnosis of pneumothorax using computed tomography (CT) scans. This research seeks to address this gap by introducing an intelligent model designed for the automatic detection of pneumothorax in chest CT images. By leveraging advanced machine learning techniques and extracting relevant radiomics features, this study aims to enhance diagnostic accuracy and efficiency. The proposed model not only aims to assist radiologists in identifying pneumothorax but also endeavors to improve clinical decision-making processes and patient outcomes by facilitating timely and accurate diagnoses. Through this innovative approach, we hope to contribute significantly to the field of medical imaging and artificial intelligence in healthcare.

## 2 Materials and methods

**Fig 1:**
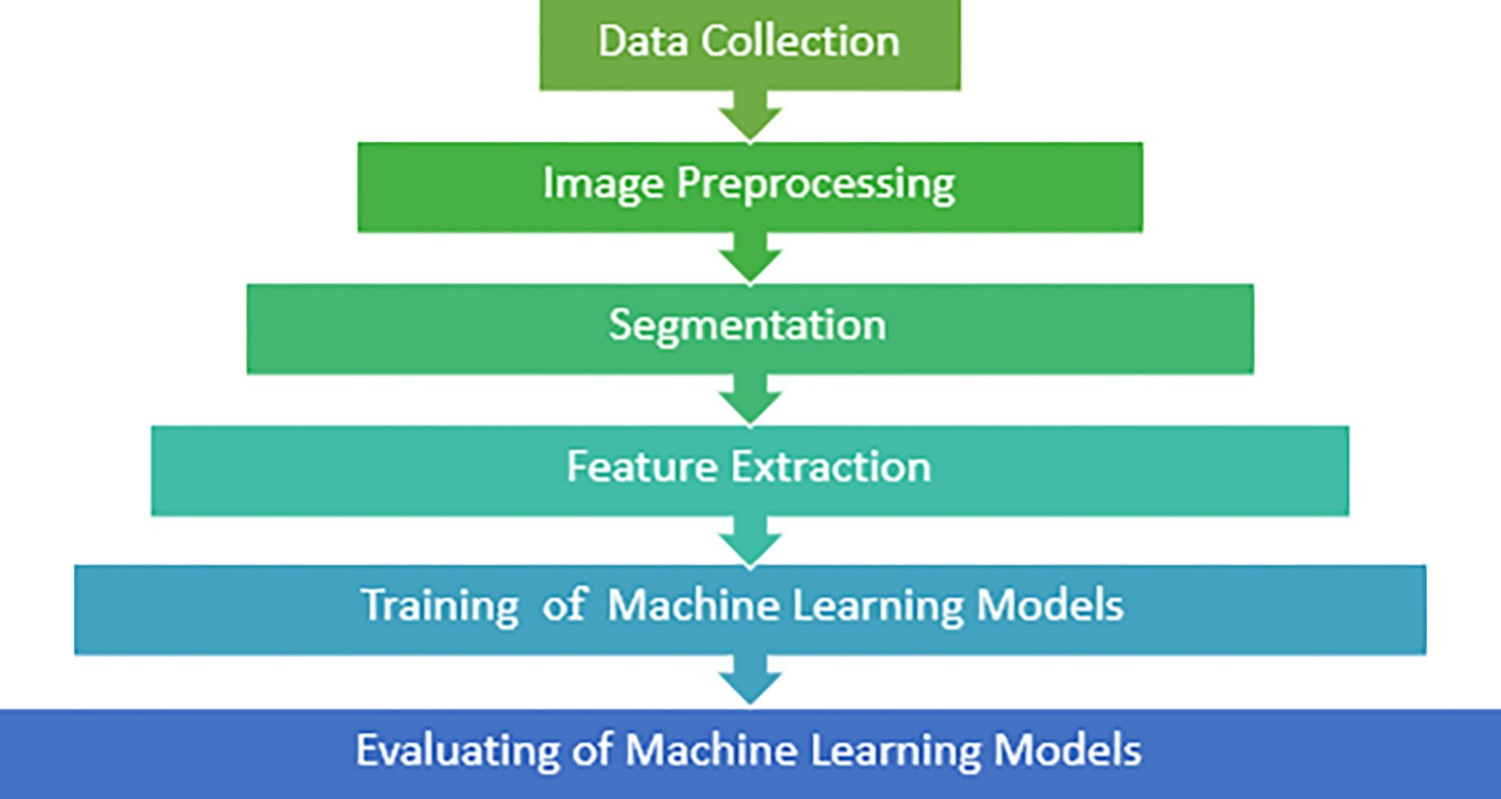
The outline of the research.

The outline of the research is shown in Fig 1.

### 2.1 Study design and settings

This was a retrospective, cross-sectional study conducted at Imam Reza Hospital in Kermanshah city, Iran. The study included patients admitted to the hospital with suspected pneumothorax over a two-year period from October 23, 2021, to October 23, 2023.

#### 2.1.1 Study population

The study population consisted of 175 patients, of which 75 were diagnosed with pneumothorax and 100 were without pneumothorax. The average age of the subjects was 55.35 ± 19.44 years, with 58 women (34%) and 117 men (66%). Detailed information about patients with pneumothorax is shown in Table 1.

**Table 1:** Characteristics of patients with pneumothorax.

Characteristic	Mean or Frequency
age	50.35±14.54*y
sex	
male	54
female	21
chest pain	21
shortness of breath	57
other lung diseases	52
trauma	26
Severity of pneumothorax	
Mild (< 2 cm)	20
Medium (2–4 cm)	31
Severe (˃ 4 cm)	24

*Standard deviation = 14.54.

#### 2.1.2 Data collection

The data used in this research was collected from the hospital’s electronic medical records and imaging database. The data included demographic information (age and gender) and clinical parameters, such as the presence of other lung diseases (including bronchiectasis, COVID-19, emphysema, metastasis, abscess, atelectasis, pneumonia), history of trauma, shortness of breath, and chest pain. CT images of suspected pneumothorax patients were also collected.

#### 2.1.3 Ethical considerations

The study was approved by the local ethics committee, and all data were fully anonymized before being accessed by the researchers. Permission was obtained from the hospital administration to access the data. This study was performed in line with the principles of the Declaration of Helsinki. Approval was granted by the Ethics Committee of Kermanshah university of medical science (IR.KUMS.MED.REC.1401.168).

#### 2.1.4 CT imaging protocol

CT images were acquired using a 16-slice CT scanner owned by Siemens. The images were obtained at slice thicknesses of 1.5, 3, and 5 mm, tube voltages of 110, 120, and 130, and exposure ranges of 24 to 332 mAs.

### 2.2 Data pre-processing

The study utilized axial CT images as this view optimally depicts pneumothorax at different depths within the lung parenchyma. On average, one slice was selected from every five slices, resulting in the analysis of approximately 980 slices in total. The selection of slices was carefully considered to ensure comprehensive coverage of all shapes and locations of pneumothorax, ranging from the lung apex to the base. The acquired DICOM (Digital Imaging and Communications in Medicine) images were preprocessed using the lung window preset [500–1400] to enhance visualization of the lung fields.

The initial phase of this study involved essential preprocessing of the CT images to mitigate noise, reduce variations, and improve overall image quality. A systematic approach was employed, utilizing the MATLAB (MATrix LABoratory) software to apply a series of preprocessing stages. These techniques were designed to optimize image clarity, minimize artifacts, and ensure a standardized input for subsequent analyses. The thoughtful application of preprocessing is crucial in refining the dataset, strengthening the robustness of downstream analyses, and enhancing the accuracy of artificial intelligence models deployed for pneumothorax diagnosis.

The specifics of the pre-processing procedures employed in MATLAB encompassed a comprehensive array of techniques aimed at standardizing image features, improving contrast, and minimizing potential confounding factors. This methodical pre-processing step was a pivotal preparatory step in the investigation, laying the foundation for the subsequent application of machine learning models and radiomics feature extraction.

In the preprocessing of CT scan images, a bilateral filter is initially employed to effectively diminish noise and mitigate artifacts that may have been introduced during the image acquisition process. Subsequently, a histogram equalization technique is applied to enhance the sharpness and contrast of the images. This preprocessing pipeline aims to optimize the quality and visual characteristics of the CT scan images for subsequent analysis [28].

**Bilateral filters** are nonlinear edge-preserving filters with image denoising. These filters replace the intensity of each pixel with the weighted average of the intensities of neighboring pixels. These weights can be based on a Gaussian distribution [29].

**Histogram equalization** stands as a prevalent, straightforward, and efficacious method for enhancing contrast in digital images. In this technique, a luminance transformation function is derived from the histogram of the image. Application of this transformation function to the image results in an output image characterized by a uniform histogram, thereby leading to an improvement in overall contrast. This method is particularly advantageous for addressing variations in pixel intensity across an image, ensuring a more balanced and visually discernible representation. The simplicity and effectiveness of histogram equalization render it a widely employed tool in image processing, contributing to the enhancement of image quality and facilitating subsequent analyses in various applications, including medical imaging such as chest X-rays and CT scans [30].

### 2.3 Segmentation

**Fig 2:**
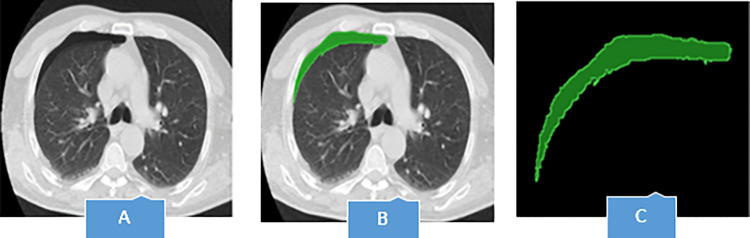
Segmentation of the pneumothorax area, the blue arrows in picture A, show air in the pleural cavity, which has caused the right lung to partially collapse.

For any radiomics approach, determination of the region of interest (ROI) is the crucial first step in the pipeline. ROIs define the region in which radiomics features are calculated [31]. After the pre-processing step, in the slices belonging to the patients, the region of pneumothorax was identified, and in the slices of healthy people (without pneumothorax), the corresponding region of the healthy lung parenchyma was identified. In this research, segmentation was done manually by two experienced radiologists, with more than 15 years of experience in thoracic imaging, and in the 3D Slicer software environment. Finally, the segmented area was cut and separated from the image. An example of segmentation of the pneumothorax region is shown in Fig 2.

### 2.4 Feature extraction and selection

In this study, this step refers to the concept of radiomics. In general, radiomics aims to extract quantitative and ideally repeatable information from diagnostic images, including complex patterns that are difficult to detect or quantify by the human eye [25]. This step was done with the Radiomics toolbox in 3D Slicer software. The extracted features include two sets of features: first-order statistical features and texture features including gray level dependence matrix (GLDM), gray level run length matrix (GLRLM), gray level size zone matrix (GLSZM), and Neighboring Gray Tone Difference Matrix (NGTDM). These features are obtained by using Wavelet filters with different decompositions (all possible combinations using a high pass or low pass filter in all three dimensions including HHH, HHL, HLH, HLL, LHH, LHL, LLH, and LLL). All these features were saved in an Excel file.

Wavelet analysis of an image is possible using a pair of square mirror filters, a high-pass filter, and a low-pass filter [32]. The high-pass filter highlights the changes in the gray level and therefore emphasizes the details of the image, while the low-pass filter smoothes the image in terms of the gray level and removes the details of the image [25].

#### First-order features

These features describe the intensity distribution of pixels or voxels in the image area defined by the mask through common and basic criteria [33].

#### Gray level dependence matrix (GLDM)

The features of this group define the gray level dependencies in an image. Gray-level dependency is defined as the number of related voxels in a certain distance that is dependent on the central voxel [33].

#### Gray level run length matrix (GLRLM)

These features provide information about the spatial distribution of the run of consecutive pixels with the same gray level, in one or more directions, in 2 or 3 dimensions [25].

#### Gray level size zone matrix (GLSZM)

GLSZM is based on a similar principle to GLRLM, but here, counting the number of groups (so-called regions) of contiguous adjacent pixels or voxels with the same gray level forms the basis of the matrix. A tissue with more homogeneity creates a wider and flatter matrix [33,34].

#### Neighboring gray tone difference matrix (NGTDM)

NGTDM calculates the sum of differences between the gray level of a pixel or voxel and the average gray level of pixels or voxels adjacent to it at a predetermined distance [25].

In our methodology, radiomics features are exclusively extracted from regions segmented by a qualified radiologist. For individuals with pneumothorax, only the area affected by pneumothorax is considered, while for healthy individuals (without pneumothorax), the region corresponding to the unaffected parenchyma is inputted into the 3D Slicer software for radiomics feature extraction. This meticulous segmentation by the radiologist ensures that there is no potential for overlap or interference with other pulmonary complications, enhancing the accuracy of feature extraction and analysis.

### 2.5 Training of machine learning models

To train the machine learning models, the dataset was systematically divided into two distinct subsets: training data and test data. In this study, 70% of the total dataset, comprising the selected CT image slices, was allocated for training purposes, while the remaining 30% was designated for testing the performance of the algorithms. This division is critical in supervised learning scenarios, where the model learns from labeled data to make predictions on unseen data. In supervised learning, each sample consists of two integral components: the input observations (or features] and the output observations (or labels) [35,36]. In the context of this study, the input observations are derived from radiomics features extracted from the CT images, while the output observation corresponds to the binary classification of the presence or absence of pneumothorax. The primary objective of supervised learning is to establish a functional relationship between the input features and the output labels based on the training data, enabling the model to generalize effectively to the test data [17]. The training process involved utilizing the labeled dataset, where the output variable—expertly coded by medical professionals—was incorporated into an Excel file alongside the extracted radiomics features. This structured dataset served as the foundation for training the machine learning models, allowing them to learn the underlying patterns associated with pneumothorax diagnosis. The training phase is crucial, as it enables the models to adjust their parameters to minimize prediction errors based on the training data, thereby enhancing their predictive capabilities. The evaluation of the models’ performance was conducted using the test dataset, which was not exposed to the models during training. This approach ensures that the assessment of the models reflects their ability to generalize to new, unseen data, a fundamental aspect of machine learning. By employing this rigorous training and testing methodology, the study aimed to develop robust predictive models capable of accurately diagnosing pneumothorax from chest CT images.

In this study, we used the Radiomics Toolbox in 3D Slicer software to extract dozens of image features capturing patterns, textures, and more from the segmented areas in the chest images. Radiomics features get saved into an Excel file. Then during machine learning model training, Python calls the Excel sheets and processes them into the standard tables used to train classifiers:

df = pd. read_excel("CT.xlsx")

The function pd. read_excel () is utilized to read the contents of the Excel file named "CT.xlsx". This file contains the radiomics features extracted from the segmented regions of interest (ROIs) in the chest CT images.

Then the variables x (independent variables = Radiomics features) and y (dependent variable = Output or target) are defined as follows:

x = df.iloc[:, 1:633]

y = df.iloc[:, 633]

Independent Variables (x): The variable x is defined using the iloc method of the DataFrame, which allows for integer-location based indexing. In this case, df. iloc[:, 1:633] selects all rows and the columns from index 1 to 632 (inclusive). This selection encompasses the radiomics features, which are the predictors used in the machine learning models. The choice of columns is based on the assumption that the first column (index 0) may contain non-feature data (such as patient identifiers or other metadata) that is not relevant for model training.

Dependent Variable (y): The variable y is defined as df. iloc[:, 633], which selects all rows from the column at index 633. This column is designated as the target variable, representing the binary classification of pneumothorax presence (1) or absence (0). This clear delineation between independent and dependent variables is crucial for supervised learning, as it establishes the relationship that the model will learn during the training process.

In this study, the radiomics features extracted from the chest CT images serve as the independent variables within the machine learning framework. These features encapsulate a wide range of quantitative measures that describe the underlying patterns, textures, and characteristics of the lung tissue as visualized in the imaging data. By analyzing these features, the models aim to discern subtle differences that may indicate the presence of pneumothorax. Conversely, the dependent variable in this analysis is the output that indicates the presence or absence of pneumothorax, which is coded in a binary format. Specifically, this output is represented as 1 for the presence of pneumothorax and 0 for its absence. This binary coding allows for straightforward classification, enabling the machine learning algorithms to learn the relationship between the independent variables (the radiomics features) and the outcome (the diagnosis of pneumothorax). The binary nature of the dependent variable is particularly suitable for classification tasks, as it simplifies the modeling process and facilitates the application of various machine learning algorithms designed for binary outcomes. By establishing this clear distinction between independent and dependent variables, the study aims to develop robust predictive models that can accurately classify new cases based on the extracted radiomics features, ultimately enhancing diagnostic capabilities in clinical settings. This structured approach not only aids in the effective training of the machine learning models but also contributes to the interpretability of the results, allowing for meaningful insights into the factors that influence the diagnosis of pneumothorax from chest CT images.

In this study, a total of 27 out of 30 initially tested machine learning models yielded reportable results for the classification of pneumothorax, as summarized in Table 2, which details the performance metrics based on the area under the Receiver Operating Characteristic (ROC) curve (AUC). The ROC curve is a graphical representation of a model’s diagnostic ability, plotting the true positive rate against the false positive rate at various threshold settings. The AUC provides a single scalar value that summarizes the model’s performance, with higher values indicating better discriminatory power. A comparative evaluation of these models revealed that Gradient Tree Boosting (GBM), eXtreme Gradient Boosting (XGBoost), and Light GBM (LGBM) outperformed the other algorithms across the evaluated criteria, with a particular emphasis on the AUC metric presented in the results. These models demonstrated superior performance characteristics, indicating their effectiveness in accurately classifying the presence or absence of pneumothorax based on the extracted radiomics features. Given their state-of-the-art performance during the comprehensive model screening phase, these three models were selected for more in-depth performance assessment on the dataset. The decision to focus on GBM, XGBoost, and LGBM was informed by their ability to capture complex relationships within the data and their robustness in handling the intricacies of medical imaging data. The subsequent analysis aimed to further evaluate their predictive accuracy, sensitivity, specificity, and overall clinical applicability in the context of pneumothorax diagnosis. This rigorous model selection process underscores the importance of employing advanced machine learning techniques to enhance diagnostic accuracy and improve clinical outcomes in the detection of pneumothorax from chest CT images. The findings from this study contribute to the growing body of evidence supporting the integration of machine learning models in radiological practice, ultimately facilitating earlier and more accurate diagnoses in clinical settings.

**Table 2:** The results of implementing machine learning models.

Model name	AUC	Model name	AUC
Logistic Regression	95%	SGD Classifier	97%
eXtreme Gradient Boosting	97%	Random Forest Classifier	97%
Calibrated Classifier CV	96%	SVC	96%
Passive Aggressive Classifier	96%	KNeighbors Classifier	96%
AdaBoost Classifier	95%	Bernoulli NB	93%
Linear SVC	96%	Extra Tree Classifier	92%
Decision Tree Classifier	97%	Linear Discriminant Analysis	87%
Bagging Classifier	94%	Nearest Centroid	75%
LGBM Classifier	95%	Gaussian NB	63%
Ridge Classifier CV	94%	Quadratic Discriminant Analysis	62%
Ridge Classifier	94%	Label Propagation	58%
Perceptron	93%	Label Spreading	58%
Extra Trees Classifier	97%	Dummy Classifier	50%
Gradient Tree Boosting	98%		

Although some models in Table 2 scored higher in AUC, GBM, XGBoost, and LGBM were ultimately selected as superior candidates based on more well-rounded performance using accuracy, sensitivity, and other metrics too. Furthermore, these models are considered novel as they obviate the need for a distinct feature selection step. This process is seamlessly integrated into the algorithm’s execution and increases efficiency.

Here is a brief description of each of the models:

**GBM** is a decision tree-based machine learning method. The term "boosting" refers to a special type of algorithm where weak prediction trees are combined to create a stronger prediction. In the conventional GBM method, simple and weak prediction trees are iteratively constructed and added to the prediction machine so that the prediction matches the real data well [37].

**XGBoost** is an extension based on GBM. Its superior performance has been demonstrated in many data science competitions, and its multi-core algorithms allow multiple tasks to be executed simultaneously, enabling the algorithm to scale to large data sets [38,39].

**LGBM** is a high-speed, distributed, high-performance machine learning framework based on a decision tree algorithm. This framework can be used in various tasks such as sorting, classification, regression, and other machine learning tasks. By maintaining accuracy, the speed of this framework increases about ten times, and the amount of occupied memory is about three times less. This framework has advantages such as high training efficiency, low memory occupancy, high precision, and support for parallelization, and it can also be implemented using GPU to process large data [38].

In the course of this study, a dedicated feature selection stage was not incorporated into our methodology. An intrinsic advantage of the employed machine learning models lies in their capacity to perform feature selection seamlessly during algorithm execution. This eliminates the necessity for a discrete step labeled ’feature selection.’ The most important features selected by all three models are shown in Table 3.

**Table 3:** The most important features selected by all three models in CT scan images.

GBM	XGBoost	LGBM
***First order*** *Root Mean Squared (LLL)* *10Percentile (LLL)* *10Percentile* *Median* *Median (LLH)* *90Percentile (LLL-f)* *Median (LLL)* ***GLDM*** *Large Dependence Low Gray Level Emphasis* *Low Gray Level Emphasis (LLH)* *Dependence Non Uniformity (HHL)* *Dependence Variance (LLL)* ***GLRLM*** *Run Length Non Uniformity (HHH)* ***GLSZM*** *Size Zone Non Uniformity (HHH)* *Large Area Low Gray Level Emphasis* *Large Area Low Gray Level Emphasis (HLL)* *Large Area Emphasis (LLL)* *Size Zone Non Uniformity (LLL)* ***NGTDM*** *strength* *strength (LLL)* *Busyness (LHH)*	***First order*** *10Percentile* *Median* *10Percentile (HLL)* *Entropy (HLH)* ***GLDM*** *Dependence Variance* *Gray Level Non Uniformity* *Dependence Non Uniformity (LLH)* *Small Dependence Low Gray Level Emphasis (LHL)* ***GLSZM*** *Large Area Low Gray Level Emphasis (LHL)* *Large Area Low Gray Level Emphasis (HLL)* ***NGTDM*** *Strength (LHL)* *Busyness (HHL)*	**First order** 10Percentile Kurtosis (HHL) **GLDM** Dependence Non Uniformity (LLH) Small Dependence Low Gray Level Emphasis (LHL) Small Dependence Low Gray Level Emphasis (HLL) **GLRLM** Gray Level Non Uniformity (LLH) Run Length Non Uniformity **GLSZM** Large Area High Gray Level Emphasis Size Zone Non Uniformity Gray Level Non Uniformity (LHH) Gray Level Non Uniformity (HLH) **NGTDM** Coarseness (LLH) Busyness (o-ng)

Since these features have shown their importance in the diagnosis of pneumothorax, they can be considered good candidates for biomarkers of pneumothorax complications, so it is suggested that in future studies, the correlation between these features with clinical parameters such as increased resonance, decreased lung sounds, increased respiratory rate, cyanosis, subcutaneous emphysema, etc, be investigated.

## 3 Results

The confusion matrix is a powerful tool for evaluating classification model performance and identifying areas where the model might need improvement. It provides a more detailed understanding of how well the model is performing across different classes.

In this research, in order to evaluate the performance of machine learning models, the evaluation criteria of confusion matrix including accuracy, precision, F1 score, specificity, sensitivity, as well as the area under the ROC curve (AUC) and misclassification were used. Relations 1 to 6 show how to calculate these criteria [40,41].

**Table:** 

1. Accuracy:	$\frac{TP+TN}{TP+TN+FP+FN}$
2. Precision:	$\frac{TP}{TP+FP}$
3. Sensitivity:	$\frac{TP}{TP+FN}$
4. Specificity:	$\frac{TN}{TN+FP}$
5. F1 score:	$\frac{2TP}{2TP+FP+FN}$
6. Misclassification:	1- accuracy

In our study:

✓ **TP (True positive):** Instances where the model correctly predicts the presence of pneumothorax in samples that have pneumothorax
✓ **TN (True negative):** Instances where the model correctly predicts the absence of pneumothorax in samples that do not have pneumothorax.
✓ **FP (False positive):** Instances where the model incorrectly predicts the presence of pneumothorax in samples that do not have pneumothorax.
✓ **FN (False negative):** Instances where the model incorrectly predicts the absence of pneumothorax in samples that have pneumothorax.

1. **Accuracy:** This metric measures the overall correctness of the model by considering both true positives and true negatives about all predictions.
2. **Precision:** Precision assesses the proportion of true positive predictions among all positive predictions, emphasizing the model’s ability to avoid false positives.
3. **F1 Score:** The F1 score is the harmonic mean of precision and recall, providing a balance between precision and sensitivity (recall).
4. **Specificity (True Negative Rate):** This metric evaluates the proportion of actual negatives correctly predicted by the model, complementing sensitivity.
5. **Sensitivity (Recall or True Positive Rate):** Sensitivity measures the proportion of actual positives correctly predicted by the model, highlighting its ability to capture positive instances.
6. **Area Under the ROC Curve (AUC):** The ROC curve plots the true positive rate against the false positive rate at various thresholds. AUC quantifies the overall discriminatory power of the model, with a higher AUC indicating better performance.
7. **Misclassification Rate:** This metric calculates the overall rate of misclassified instances, encompassing both false positives and false negatives [42].

**Fig 3:**
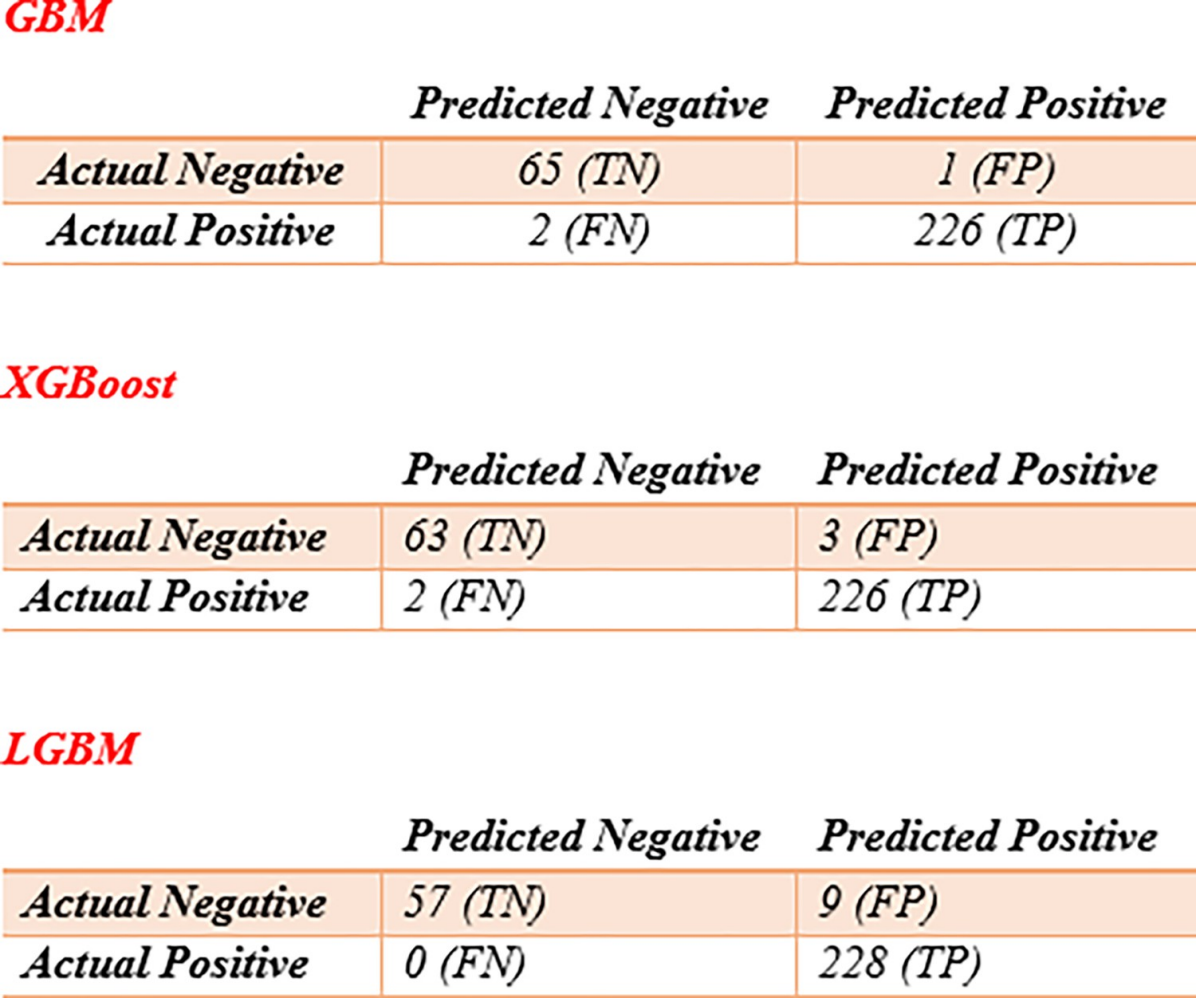
The confusion matrix for GBM, XGBoost and LGBM.

The confusion matrix for all three models is shown in Fig 3.

The calculated evaluation criteria for all models are shown in Table 4.

**Table 4:** The results of implementing the models.

Models	Accuracy	Precision	AUC	Mis-Classification	Sensitivity	Specificity	F1- Score
**GBM**	98.97%	99.55%	98.80%	1.02%	99%	98%	99%
**XGBoost**	98.29%	98.68%	97.28%	1.70%	99%	95%	97%
**LGBM**	96.93%	96.20%	93.18%	3.06%	100%	86%	93%

Table 4 presents the performance metrics of the machine learning models evaluated in this study. The Gradient Boosting Machine (GBM) model demonstrated the highest accuracy, achieving a value of 98.97%, followed closely by the XGBoost model at 98.29%.

In terms of precision, the GBM model outperformed the other models, recording a precision score of 99.55%. Regarding sensitivity, all three models—GBM, XGBoost, and LightGBM (LGBM)—exhibited strong performance, with sensitivity values of 99%, 99%, and 100%, respectively, indicating their effectiveness in correctly identifying positive cases.

For specificity, the GBM model achieved a score of 98%, while the XGBoost and LGBM models recorded specificity values of 95% and 86%, respectively. This indicates that the GBM model has a superior ability to correctly identify negative cases compared to the other models.

The F1 score, which balances precision and recall, was highest for the GBM model at 99%, followed by the XGBoost model at 97% and the LGBM model at 93%, further confirming the GBM model’s robustness in precision.

The misclassification rate, representing the proportion of incorrectly classified cases, was lowest for the GBM model at 1.02%, with the XGBoost model following at 1.70%. Finally, in terms of the Area Under the Receiver Operating Characteristic Curve (AUC), both the GBM and XGBoost models outperformed the LGBM model, achieving AUC values of 98.80% and 97.28%, respectively.

These results underscore the superior performance of the GBM model across multiple evaluation criteria, highlighting its potential utility in the clinical diagnosis of pneumothorax from chest CT scan images.

## 4 Discussion

This study demonstrates the effectiveness of machine learning models in accurately detecting pneumothorax from chest CT scan images, highlighting the potential of these technologies to enhance diagnostic accuracy in emergency medicine. Our models achieved impressive performance metrics, particularly in terms of Sensitivity. By incorporating radiomics features, we were able to capture intricate details from the CT images, which contributed to improved classification performance. These findings underscore the feasibility of integrating machine learning techniques into clinical workflows to facilitate timely diagnosis and intervention for patients presenting with pneumothorax.

One of the key strengths of our study is the use of radiomics features in the machine learning models. Previous research on pneumothorax diagnosis using machine learning did not explore the potential of radiomics, making our approach novel and distinctive. By incorporating radiomics features, we were able to capture detailed characteristics of the CT images, potentially enhancing the models’ ability to distinguish between normal and abnormal findings. Another strength of our study is the careful selection and preprocessing of the training data. We ensured that the training and test datasets had the same distribution, minimizing bias and improving the reliability of our results. This attention to data quality is crucial for developing robust machine learning models that can generalize well to new, unseen data.

While our study demonstrates promising results in the diagnosis of pneumothorax using machine learning, there are several limitations that should be acknowledged:

One significant limitation is the lack of comparative studies using radiomics features for pneumothorax diagnosis. This makes it challenging to evaluate our models’ performance against other approaches that utilize similar methodologies. Additionally, while our models demonstrated strong overall performance, a direct comparison with deep learning techniques, such as the convolutional neural network (CNN) used by Li (2019) [1], reveals that deep learning models may have higher sensitivity for pneumothorax detection. Another limitation is the potential for overfitting, a common issue in machine learning. While we took steps to minimize this risk by using appropriate validation strategies, such as cross-validation, it is essential to validate the models on external datasets to ensure their generalizability. Our study was conducted at a single institution, which may limit the generalizability of our findings to other populations and healthcare settings. Future research should aim to validate our models using data from multiple centers to ensure robustness. As a retrospective study, we relied on previously collected data, which may be subject to selection bias and incomplete information. Prospective studies are needed to confirm the clinical utility of our approach in real-world settings. Our study focused primarily on radiomics features derived from CT images, without incorporating additional clinical variables that may influence pneumothorax diagnosis, such as patient symptoms, medical history, and laboratory findings. Future studies should explore the integration of these factors to further enhance diagnostic performance. Our study did not assess the long-term outcomes of patients diagnosed with pneumothorax using our machine learning models. Evaluating the impact of our approach on patient management and prognosis is an important area for future research.

Future research should focus on addressing these limitations and further exploring the potential of machine learning in pneumothorax diagnosis. Collaborative efforts between researchers and clinicians will be crucial for developing and validating machine learning models that can be seamlessly integrated into clinical workflows. Additionally, investigating the interpretability and explainability of these models will be important for building trust and acceptance among healthcare professionals. By addressing these limitations and exploring future research directions, we believe our study can contribute to the development of more accurate and clinically relevant tools for the diagnosis of pneumothorax using machine learning and radiomics techniques.

In broad terms, the efficacy and efficiency of a machine learning model are contingent upon the inherent nature and attributes of the data, coupled with the proficiency of the learning algorithm. As a consequence, the performance outcomes of diverse artificial intelligence models, encompassing both machine learning and deep learning, exhibit a high degree of reliance on the specifics of the training data. The application of machine learning models to distinct datasets yields disparate results, highlighting the challenge of selecting an optimal learning algorithm for a given target application in certain domains [43]. The difficulty arises from the fact that each learning algorithm pursues distinct objectives, and even within the same category, outcomes may diverge based on the inherent characteristics of the data [44].

In a study conducted in 2021 by Rachel Lea Draelos et al. [45] to evaluate chest CT scan images for several common pathologies using machine learning techniques, the AUC value when considering 9 pathologies was equal to 81.6%, and when 83 pathologies were investigated, it was calculated equal to 90.4%. Although the study [23] showed that training the model using more labels leads to better performance of the model, all three models used in our research had better performance in terms of AUC.

In a retrospective study conducted in 2019 by Xiang Li [1] at Massachusetts General Hospital, an eight-layer convolutional neural network was trained using fixed-size 2D images on 80 chest CT scans. The performance of the CNN program was evaluated on 200 chest CT scans. Subjective sensitivity was 100% and specificity was 82.5%. This program based on deep learning showed high sensitivity for the automatic detection of pneumothorax in chest CT scans [1], But all the models used in our study showed a stronger performance in terms of specificity criteria.

Although the sensitivity in the study [1] using deep learning models is higher, deep learning is a resource-intensive technology. To train the models, high-performance GPUs, a lot of storage space, etc. are needed, which makes the machine-learning techniques used in our research simpler and less expensive.

Our findings demonstrate that the machine learning models employed in this study achieved impressive performance metrics, particularly in terms of AUC. This is noteworthy when compared to other studies, such as the work by Draelos et al. (2021), which found AUC values of 81.6% for nine pathologies and 90.4% for 83 pathologies. While their study suggests that increasing the number of pathology labels can improve model performance, our models outperformed these benchmarks.

## 5 Conclusion

The integration of artificial intelligence and machine learning in emergency medicine offers significant potential to tackle critical challenges, particularly in disease diagnosis and patient triage. Timely identification of emergency conditions, such as pneumothorax, is essential for facilitating prompt interventions in the emergency department (ED) and preventing further complications. Our research demonstrates the effectiveness of machine learning models in accurately detecting pneumothorax from CT scan images, highlighting their potential to enhance diagnostic precision. The practical implications of our findings suggest that deploying automated detection systems can serve as a rapid and reliable solution for diagnosing pneumothorax in emergency settings. The ease of integrating machine learning techniques, coupled with the impressive performance metrics observed in our study and corroborated by other research, underscores the viability of these methods. By establishing robust systems for distinguishing between patients with and without pneumothorax, healthcare developers can significantly improve patient outcomes in radiology departments. Ultimately, our study advocates for the adoption of machine learning technologies as a transformative approach to enhance diagnostic capabilities and streamline emergency care processes.
