# Correction: Radiomics-based machine learning for automated detection of Pneumothorax in CT scans

**DOI:** 10.1371/journal.pone.0321698

**Published:** 2025-04-01

**Authors:** Hanieh Alimiri Dehbaghi, Karim Khoshgard, Hamid Sharini, Samira Jafari Khairabadi, Farhad Naleini

There are errors in the author affiliations. The correct affiliations are as follows:

Hanieh Alimiri Dehbaghi^1^, Karim Khoshgard^1^, Hamid Sharini^2^, Samira Jafari Khairabadi^3^, Farhad Naleini^4^

**1** Department of Medical Physics, Kermanshah University of Medical Sciences, Kermanshah, Iran, **2** Department of Biomedical Engineering, Kermanshah University of Medical Sciences, Kermanshah, Iran, **3** Student Research Committee, Kermanshah University of Medical Sciences, Kermanshah, Iran, **4** Clinical Research Development Center, Imam Reza Hospital, Kermanshah University of Medical Sciences, Kermanshah, Iran.
